# Effect of pre-operative internal obturator muscle mass index in MRI on biochemical recurrence of prostate cancer patients after radical prostatectomy: a multi-center study

**DOI:** 10.1186/s12894-021-00853-0

**Published:** 2021-05-26

**Authors:** Won Tae Kim, Ho Won Kang, Sung Pil Seo, Yong-June Kim, Sang Cheol Lee, Wun-Jae Kim, Bum Sang Cho, Yun Sok Ha, Tae Gyun Kwon, Jinsung Park, Seung Chol Park, Young Beom Jeong, Taek Won Kang, Sung-Woo Park, Seok Joong Yun

**Affiliations:** 1 Department of Urology, College of Medicine, Chungbuk National University, 62, Kaeshin-dong, Heungduk-ku, Cheongju, Chungbuk 361-711 South Korea; 2 Department of Urology, Chungbuk National University Hospital, Cheongju, South Korea; 3 Department of Radiology, College of Medicine, Chungbuk National University, Cheongju, South Korea; 4 Department of Urology, College of Medicine, Kyungpook National University, Daegu, South Korea; 5 Department of Urology, College of Medicine, Eulji University, Daejeon, South Korea; 6 Department of Urology, College of Medicine, Won Kang University, Iksan, South Korea; 7 Department of Urology, College of Medicine, Jeonbuk National University, Jeonju, South Korea; 8 Department of Urology, College of Medicine, Jeonnam National University, Kwangju, South Korea; 9 Department of Urology, College of Medicine, Pusan National University, Pusan, South Korea

**Keywords:** Prostatic neoplasms, Sarcopenia, Internal obturator muscle, Recurrence

## Abstract

**Background:**

Recent reports show that the pre-operative or post-operative skeletal mass index (sarcopenia) affects survival rates for various cancers; however, the link between prostate cancer survival and sarcopenia is unclear. Therefore, this study examined the effect of the pre-operative internal obturator muscle (IOM) mass index on biochemical recurrence (BCR) of prostate cancer (PCa) patients who underwent radical prostatectomy.

**Methods:**

In total, 222 patients, who underwent open, laparoscopic, or robot-assisted radical prostatectomy at seven centers in 2011 and were followed up for 5 years, were enrolled. BCR was examined in the context of pre-operative IOM mass index and BMI.

**Results:**

The mean age of the patients was 67.82 ± 6.23 years, and the mean pre-operative prostate-specific antigen (PSA) level was 11.61 ± 13.22 ng/ml. There was no significant difference in baseline characteristics between the low and high IOM mass index groups (*p* > 0.05). Age, pre-op PSA level, ECE, and T-stage were associated with BCR (*p* = 0.049, *p* < 0.001, *p* = 0.001, *p* = 0.004, respectively). BMI, prostate volume, Gleason score, resection margin, N-stage, M-stage and IOM mass index was not associated with BCR (*p* > 0.05).

**Conclusions:**

Pre-operative IOM mass index was not associated with BCR; however, long-term follow-up is necessary to evaluate cancer-specific and overall survival of PCa patients.

## Background

Prostate cancer (PCa) is the most common cancer in men worldwide [1]. The incidence of PCa in Asia is among the lowest in the world [1]. However, the incidence in the Republic (Rep.) of Korea is increasing rapidly [2]. PCa mortality rates have fallen in high-resource countries, but have increased or remain stable in low-resource countries [1]. PCa-associated mortality in the Rep. of Korea is increasing along with incidence [2].

Sarcopenia is a condition characterized by progressive and generalized loss of muscle mass and strength [3]. Sarcopenia predicts drug toxicity and time-to-tumor progression in patients undergoing chemotherapy [4]. The impact of sarcopenia in cancer patients has been studied, and data suggest that sarcopenia is independently associated with post-operative outcome following resection of colorectal cancer, hepatocellular carcinoma, pancreatic cancer, and bladder cancer [5–8]. In addition, sarcopenia is a prognostic marker for disease recurrence and mortality in patients with urologic cancers [8–11].

Radical prostatectomy (RP) is the gold standard treatment for localized PCa. RP is often the treatment of choice for younger and less morbid patients. A previous report suggests that sarcopenia is associated with non-cancer-related death in PCa patients undergoing radiotherapy [12]. Conversely, Mason et al. report that sarcopenia is not independently associated with perioperative complications or oncologic outcomes after RP [13]. Therefore, there is a lack of evidence supporting an association between sarcopenia and oncologic outcomes in PCa. Generally, sarcopenia, skeletal mass index (SMI) was defined as the cross-sectional area of the rectus abdominis; internal, external, and transverse obliques; psoas; quadratus lumborum; and erector spinae muscles from L3 down. Most surgeon performed MRI imaging work-up for prostatectomy. However, preoperative CT imaging is the option and surgeon’s preference. Unfortunately, cross sectional area of whole muscle from L3 down level cannot be taken in the MRI imaging. Internal obturator muscle (IOM) is the only measurable muscle in the pelvis MRI axial cut. Additionally, there was no study between pre-operative IOM mass index and oncologic outcomes in PCa. Therefore, we investigated the effect of pre-operative IOM mass index on biochemical recurrence (BCR) in Korean patients after RP.

## Methods

### Study population and data collection

In total, 222 PCa patients, who underwent open, laparoscopic, or robot-assisted RP at seven academic centers in 2011 and were followed up for 5 years, were enrolled. All patients had histologically confirmed primary adenocarcinoma of the prostate, and all had undergone pre-operative MRI within 3 months of surgery. Patients were excluded if they had previously received androgen deprivation therapy (ADT) or radiotherapy (RT). In addition, patients undergoing testosterone replacement therapy or taking medications that affect muscle mass were excluded.

**Fig. 1:**
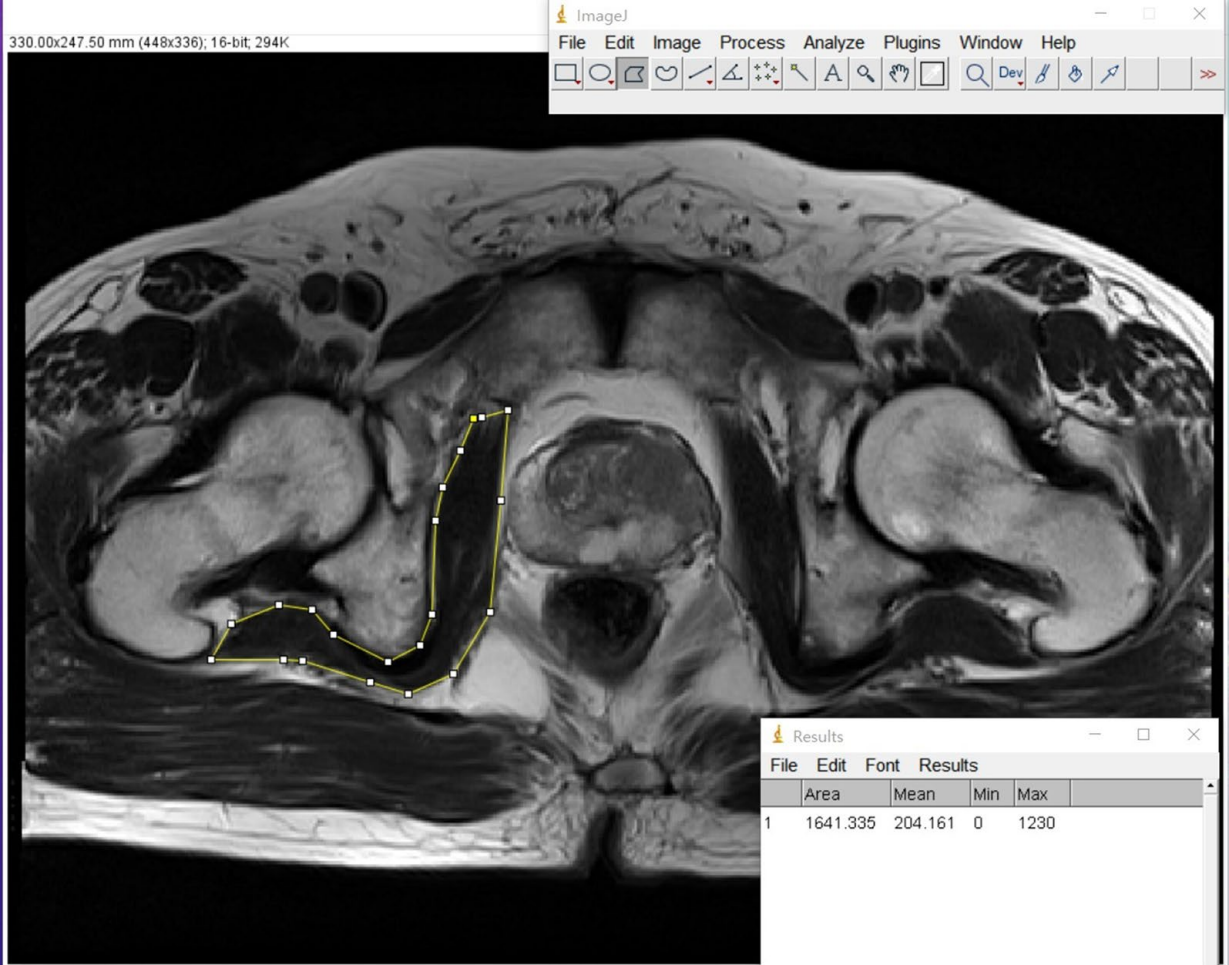
The measuring methods of internal obturator muscle width in the pelvis MRI for IOM mass index

Clinical and pathologic data included age, height, body weight, prostate-specific antigen (PSA) levels, prostate volume, pathologic T-, N-, and M-stage, receipt of adjuvant RT or ADT, and follow-up duration. BCR after RP was defined as a post-operative PSA level > 0.2 ng/mL. Patients undergoing adjuvant ADT or RT after RP were excluded in the BCR group. IOM mass index was measured by dividing the mean internal obturator muscle area (calculated from MRI) by height (squared). All internal obturator muscle area measurements were performed by a single radiologist using ImageJ (https://imagej.nih.gov/ij/) at the level of symphysis pubis (Cho, BS, Fig. 1). Median value of IOM mass index was used to determine the cutoff value between high and low IOM mass index. Using this median value (11.47708194 cm^2^/m^2^), patients were classified into high or low IOM mass index group. BMI group were divided into 3 groups according to Korean BMI criteria (normal < 23 kg/m^2^; 23 kg/m^2^ ≤ overweight < 25 kg/m^2^; 25 kg/m^2^ ≤ obese).

### Statistical analysis

IOM mass index was divided into two groups according to a median cut-off value. BMI was divided to three groups according to Korean BMI criteria [14]. The baseline characteristics of the patients with a low IOM mass index and a high IOM mass index were compared using an independent t-test and the Chi-square test as appropriate. Comparisons of clinical and pathological parameters according to BMI were made using ANOVA and the Chi-square test as appropriate. The association between BCR and pre-operative IOM mass index or BMI values were examined using a Cox proportional hazards model and a Kaplan–Meier survival analysis. Statistical analyses were performed using the Statistical Package for Social Sciences, version 25 software (SPSS, Inc., Chicago, IL, USA). A p-value < 0.05 was considered statistically significant.

## Results

### Baseline characteristics of the study population according to IOM mass index

The baseline characteristics of all the patients are shown in Table 1. The mean age was 67.82 ± 6.23 years, and the mean pre-operative PSA level was 11.61 ± 13.22 ng/mL. There was no significant difference in baseline characteristics between the low IOM mass index and high IOM mass index groups (*p* > 0.05).

**Table 1:** Baseline characteristics of all patients and according to the Internal obturator muscle (IOM) mass Index

Parameters	Overall	According to the IOM mass index		
		Low IOM	High IOM	*p*-value
Number	222	111	111	
Age (years)	67.82 ± 6.23	68.55 ± 6.11	67.10 ± 6.29	0.083
IOM (mm^2^/cm^2^)	11.23 ± 2.22			
BMI (kg/m^2^)	24.46 ± 2.86	24.17 ± 2.86	24.74 ± 2.85	0.136
Pre-op PSA (ng/mL)	11.61 ± 13.22	11.12 ± 12.29	12.11 ± 14.12	0.579
Prostate volume (cc)	34.58 ± 14.13	35.56 ± 15.68	33.57 ± 12.33	0.313
*Type of operation*				0.200
RRP	163 (73.4)	77 (69.4)	86 (77.5)	
LRP	1 (0.5)	0	1 (0.9)	
RARP	58 (26.1)	34 (30.6)	24 (21.6)	
*Gleason score*				0.441
≤ 6	59 (26.6)	24 (21.6)	35 (26.6)	
7	145 (65.3)	78 (70.3)	67 (65.3)	
8	8 (3.6)	4 (3.6)	4 (3.6)	
9	10 (4.5)	5 (4.5)	5 (4.5)	
*T-stage*				0.717
T2a	28 (12.6)	14 (12.6)	14 (12.6)	
T2b	38 (17.1)	19 (17.1)	19 (17.1)	
T2c	119 (53.6)	56 (50.5)	63 (56.8)	
T3a	21 (9.5)	13 (11.7)	8 (7.2)	
T3b	16 (7.2)	9 (8.1)	7 (6.3)	
*N-stage*				0.155
0	267 (97.1)	111 (100)	109 (98.2)	
1	8 (2.9)	0 (0.0)	2 (1.8)	
*cM stage*				0.316
0	221 (99.5)	110 (99.1)	111 (100)	
1	1 (0.5)	1 (0.9)	0 (0.0)	
*BCR*				0.871
0	173 (77.9)	87 (78.4)	86 (77.5)	
1	49 (22.1)	24 (21.6)	25 (22.5)	

BMI, body mass index; pre-op PSA, pre-operative prostate-specific antigen; RRP, radical retropubic prostatectomy; LRP, laparoscopic radical prostatectomy; RARP, robot-assisted radical prostatectomy; BCR, biochemical recurrence

### Comparison of clinical and pathological parameters according to BMI

As shown in Table 2, there was no significant difference in clinical and pathological parameters according to BMI (*p* > 0.05).

**Table 2:** Comparison of clinical and pathological parameters according to body mass index

Parameter	≤ Normal	Overweight	Obese	*p*-value
Number	63	73	86	
Age (years)	68.57 ± 6.06	68.07 ± 6.85	67.07 ± 5.76	0.321
IOM (mm^2^/cm^2^)	10.84 ± 2.18	11.13 ± 2.02	11.59 ± 2.37	0.115
BMI (kg/m^2^)	21.11 ± 1.45	24.06 ± 0.51	27.25 ± 1.79	< 0.001
Pre-op PSA (ng/mL)	13.97 ± 17.97	11.01 ± 10.93	10.40 ± 10.57	0.237
Prostate volume (cc)	32.54 ± 12.95	33.01 ± 14.39	37.54 ± 14.42	0.062
*Type of operation*				0.291
RRP	48 (76.2)	58 (79.5)	57 (66.3)	
LRP	0 (0.0)	0 (0.0)	1 (1.2)	
RARP	15 (23.8)	15 (20.5)	28 (32.6)	
*Gleason score*				0.165
≤ 6	14 (22.2)	20 (27.4)	25 (29.1)	
7	43 (68.3)	49 (67.1)	53 (61.6)	
8	5 (7.9)	1 (1.4)	2 (2.3)	
9	1 (1.6)	3 (4.1)	6 (7.0)	
*T-stage*				0.220
T2a	7 (11.1)	14 (19.2)	7 (8.1)	
T2b	7 (11.1)	13 (17.8)	18 (20.9)	
T2c	37 (58.7)	37 (50.7)	45 (52.3)	
T3a	5 (7.9)	5 (6.8)	11 (12.8)	
T3b	7 (11.1)	4 (5.5)	5 (5.8)	
*N-stage*				0.524
0	62 (98.4)	72 (98.6)	86 (100)	
1	1 (1.6)	1 (1.4)	0 (0.0)	
*cM stage*				0.282
0	62 (98.4)	73 (100)	86 (100.0)	
1	1 (1.6)	0 (0.0)	0 (0.0)	
*BCR*				0.521
0	46 (73.0)	59 (80.8)	68 (79.1)	
1	17 (27.0)	14 (19.2)	18 (20.9)	

IOM, Internal obturator muscle mass index; BMI, body mass index; pre-op PSA, pre-operative prostate-specific antigen; RRP, radical retropubic prostatectomy; LRP, laparoscopic radical prostatectomy; RARP, robot-assisted radical prostatectomy; BCR, biochemical recurrence

### Cox proportional hazards model analysis of biochemical recurrence

**Fig. 2:**
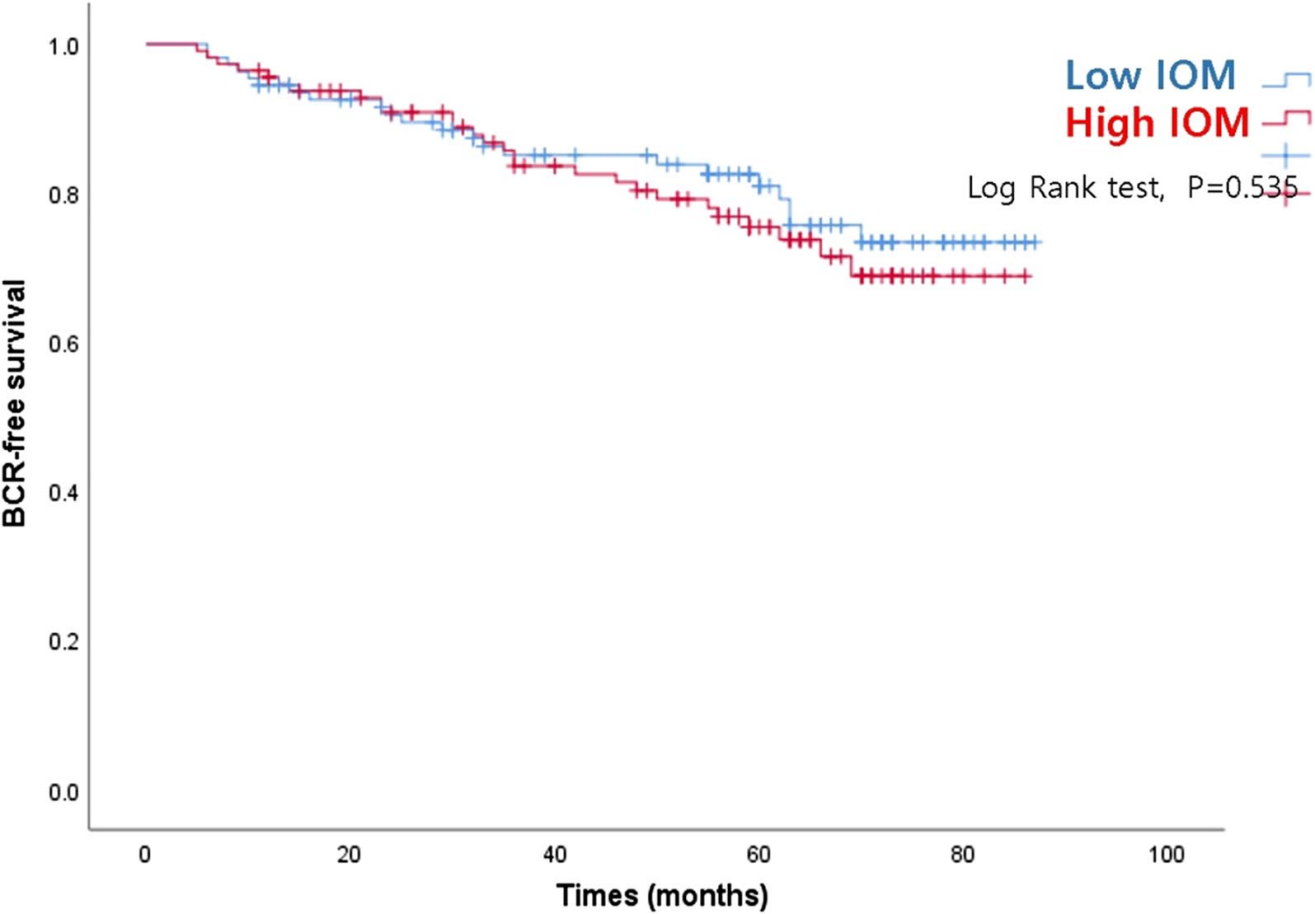
Biochemical recurrence (BCR)-free survival according to internal obturator muscle (IOM) mass index status (low IOM vs. high IOM)

Age, pre-op PSA, ECE, and T-stage was associated with BCR (*p* = 0.049, *p* < 0.001, *p* = 0.001, *p* = 0.004, respectively). However, there was no association between BCR and BMI, prostate volume, Gleason score, resection margin, N-stage, or M-stage (*p* > 0.05; Table 3). In particular, IOM mass index was not associated with BCR (*p* > 0.05; Fig. 2).

**Table 3:** Cox proportional hazards model analysis of biochemical recurrence

Parameter	HR	95% CI	*p*-value
Age (years)	0.945	0.892–1.000	0.049
IOM (mm^2^/cm^2^) (low vs. high)	0.891	0.462–1.719	0.731
*BMI* (kg/m^2^)			0.142
≤ Normal	–		
Overweight	0.880	0.379–2.042	
Obesity	0.455	0.195–1.063	
Pre-op PSA (ng/ml)	1.064	1.045–1.083	< 0.001
Prostate volume (cc)	1.016	0.990–1.042	0.226
*Gleason score*			0.364
≤ 6	–		
7	1.263	0.478–3.340	
8	1.473	0.317–6.844	
9	3.064	0.798–11.760	
ECE (no vs. yes)	5.551	2.011–15.322	0.001
Resection margin (– vs. +)	1.040	0.479–2.262	0.920
*T-stage*			0.004
T2a	–		
T2b	13.260	2.218–79.262	
T2c	3.157	0.609–16.373	
T3a	1.564	0.177–13.785	
T3b	4.830	0.700–33.326	
N-stage (no involvement vs. LN involvement)	0.300	0.028–3.202	0.319
M-stage (no meta vs. meta)	0.000	0.000–	0.985

IOM, internal obturator muscle mass index; BMI, body mass index; pre-op PSA, pre-operative prostate-specific antigen; ECE, extracapsular extension; LN, lymph node

## Discussion

The results of the present study suggest that IOM mass index is not associated with BCR after RP in Korean men. The present study is the first to show no significant association between IOM mass index and BCR in men with RP. Thus, IOM mass index may not be a prognostic marker for BCR in Korean men with localized PCa undergoing RP.

Previous studies suggest that high BMI is associated with increased risk of PCa [15, 16]. A meta-analysis by Bergstrom et al. reports a 6% increase in the risk of PCa in overweight men and a 12% increase in obese men compared with men of normal weight [16]. Maclnnis et al. reported a weak positive association between BMI and risk of PCa; BMI was associated with moderate increase in the risk of advanced PCa [15].

The relationship between BMI and BCR after RP remains controversial. Freedland et al. reported that obesity among men treated with RP was associated with high-grade tumors, a trend toward increased risk of a positive surgical margin, and high BCR [17]. Magheli et al. reported that high BMI is associated with adverse pathological findings and is a strong independent predictor of BCR after RP [18]. Asmar et al. reported that both obesity and hypertension are associated with an increased risk of BCR after RP, independent of age at the time of diagnosis or tumor pathological features [19]. By contrast, Tomaszewski et al. reported that obesity is not associated with adverse pathologic features, positive surgical margin, or BCR [20]. They suggested that their data provide evidence that obese men undergoing RP are not more likely to suffer PCa progression. In addition, Siddiqui et al. reported that obese patients appeared to have worse pathologic features at the time of prostatectomy; however, BMI did not appear to be an independent predictor of recurrence or survival after prostatectomy [21]. The present study found no association between BMI and BCR. Therefore, we believe that the relationship between BMI and BCR after RP remains unclear.

Sarcopenia is a process associated with normal aging; however, it is exacerbated by the hypercatabolic state and inflammatory responses caused by malignancy [22]. A systematic review by Joglekar et al. investigated the impact of sarcopenia on outcome following surgical resection of cancer and reported that sarcopenia is an independent prognostic factor for both complications and survival following surgical resection [23]. Sarcopenia is a more objective and comprehensive pre-operative risk factor that predicts all-cause survival for bladder cancer after radical cystectomy (RC) [9, 24]. With respect to PCa, several reports link sarcopenia and survival [12, 25]. Thus, sarcopenia could be used to predict non-cancer-related death in men with PCa after RT [12] and may be a poor prognostic factor for CRPC treated with chemotherapy [25]. However, the association between sarcopenia and survival after RP is very unclear. Only one study was reported that sarcopenia was not significantly associated with the risks of BCR, systemic progression, or all-cause mortality after RP [13]. This study is the first study to evaluate the association between IOM and prognosis in all cancer patients. IOM mass index is the only measurable muscle mass index in pelvic MRI. So, there was no options to select the muscle mass index in pelvic MRI. However, long-term follow-up studies are necessary to identify (or not) any association between sarcopenia or IOM mass index and survival.

We recognize that this study has several limitations. First, we only measured internal obturator muscle mass, but not SMI. In general, SMI was defined as the cross-sectional area of the rectus abdominis; internal, external, and transverse obliques; psoas; quadratus lumborum; and erector spinae muscles from L3 down. However, because this was a multi-institutional study, different authors have different protocols for pre-operative CT and MRI. Therefore, we selected only the internal obturator muscle area in the pelvis in the MRI, because of internal obturator muscle is the only measurable muscle in the pelvis MRI axial cut. So, we couldn't compared IOM mass with general SMI. Second, many patients who underwent RP at these institutions were excluded from the analysis due to differences in CT or MRI protocols. In some institutions, radiologists were not able to measure the internal obturator muscle at the same levels. Third, the follow-up period was too short to get an accurate picture of survival. In this case we did not examine cancer-specific or overall survival; studies may need follow-up data spanning more than 15 years to get an accurate picture of the association between IOM mass index and the survival of PCa patients.

## Conclusions

Pre-operative IOM mass index was not associated with BCR; however, long-term (> 15 years) follow-up is necessary to better answer this still controversial question.

## Data Availability

The datasets used and or analysed during the current study available from the corresponding author on reasonable request.

## References

[1] Center M, Jemal A, Lortet Tieulent J, Ward E, Ferlay J, Brawley O (2012). International variation in prostate cancer incidence and mortality rates. Eur Urol.

[2] Lee HY, Park S, Doo SW, Yang WJ, Song YS, Kim JH (2019). Trends in prostate cancer prevalence and radical prostatectomy rate according to age structural changes in South Korea between 2005 and 2015. Yonsei Med J.

[3] Cruz Jentoft AJ, Baeyens JP, Bauer JM, Boirie Y, Cederholm T, Landi F (2010). Sarcopenia: European consensus on definition and diagnosis: Report of the European Working Group on Sarcopenia in Older People. Age Ageing.

[4] Prado CM, Baracos VE, McCargar LJ, Reiman T, Mourtzakis M, Tonkin K (2009). Sarcopenia as a determinant of chemotherapy toxicity and time to tumor progression in metastatic breast cancer patients receiving capecitabine treatment. Clin Cancer Res.

[5] Miyamoto Y, Baba Y, Sakamoto Y, Ohuchi M, Tokunaga R, Kurashige J (2015). Sarcopenia is a negative prognostic factor after curative resection of colorectal cancer. Ann Surg Oncol.

[6] Harimoto N, Shirabe K, Yamashita YI, Ikegami T, Yoshizumi T, Soejima Y (2013). Sarcopenia as a predictor of prognosis in patients following hepatectomy for hepatocellular carcinoma. Br J Surg.

[7] Peng P, Hyder O, Firoozmand A, Kneuertz P, Schulick RD, Huang D (2012). Impact of sarcopenia on outcomes following resection of pancreatic adenocarcinoma. J Gastrointest Surg.

[8] Smith AB, Deal AM, Yu H, Boyd B, Matthews J, Wallen EM (2014). Sarcopenia as a predictor of complications and survival following radical cystectomy. J Urol.

[9] Psutka SP, Carrasco A, Schmit GD, Moynagh MR, Boorjian SA, Frank I (2014). Sarcopenia in patients with bladder cancer undergoing radical cystectomy: impact on cancer-specific and all-cause mortality. Cancer.

[10] Psutka SP, Boorjian SA, Moynagh MR, Schmit GD, Frank I, Carrasco A (2015). Mortality after radical cystectomy: impact of obesity versus adiposity after adjusting for skeletal muscle wasting. J Urol.

[11] Psutka SP, Boorjian SA, Moynagh MR, Schmit GD, Costello BA, Thompson RH (2016). Decreased skeletal muscle mass is associated with an increased risk of mortality after radical nephrectomy for localized renal cell cancer. J Urol.

[12] McDonald AM, Swain TA, Mayhew DL, Cardan RA, Baker CB, Harris DM (2017). CT Measures of bone mineral density and muscle mass can be used to predict noncancer death in men with prostate cancer. Radiology.

[13] Mason RJ, Boorjian SA, Bhindi B, Rangel L, Frank I, Karnes RJ (2018). The association between sarcopenia and oncologic outcomes after radical prostatectomy. Clin Genitourin Cancer.

[14] Kim MK, Lee WY, Kang JH, Kim BT, Kim SM, Kim EM (2014). 2014 clinical practice guidelines for overweight and obesity in Korea. Endocrinol Metab.

[15] MacInnis RJ, English DR (2006). Body size and composition and prostate cancer risk: systematic review and meta-regression analysis. Cancer Causes Control.

[16] Bergström A, Pisani P, Tenet V, Wolk A, Adami HO (2001). Overweight as an avoidable cause of cancer in Europe. Int J Cancer.

[17] Freedland SJ, Aronson WJ, Kane CJ, Presti JC, Amling CL, Elashoff D (2004). Impact of obesity on biochemical control after radical prostatectomy for clinically localized prostate cancer: a report by the Shared Equal Access Regional Cancer Hospital database study group. J Clin Oncol.

[18] Magheli A, Rais Bahrami S, Trock BJ, Humphreys EB, Partin AW, Han M (2008). Impact of body mass index on biochemical recurrence rates after radical prostatectomy: an analysis utilizing propensity score matching. Urology (Ridgewood, NJ).

[19] Asmar R, Beebe Dimmer JL, Korgavkar K, Keele GR, Cooney KA (2013). Hypertension, obesity and prostate cancer biochemical recurrence after radical prostatectomy. Prostate Cancer Prostatic Dis.

[20] Tomaszewski J, Chen Y, Bertolet M, Ristau BT, Woldemichael E, Nelson JB (2013). Obesity is not associated with aggressive pathologic features or biochemical recurrence after radical prostatectomy. Urology (Ridgewood, NJ).

[21] Siddiqui SA, Inman BA, Sengupta S, Slezak JM, Bergstralh EJ, Leibovich BC (2006). Obesity and survival after radical prostatectomy: A 10-year prospective cohort study. Cancer.

[22] Prado CM, Wells JC, Smith SR, Stephan BC, Siervo M (2012). Sarcopenic obesity: a critical appraisal of the current evidence. Clin Nutr (Edinburgh).

[23] Joglekar S, Nau PN, Mezhir J (2015). The impact of sarcopenia on survival and complications in surgical oncology: a review of the current literature. J Surg Oncol.

[24] Hirasawa Y, Nakashima J, Yunaiyama D, Sugihara T, Gondo T, Nakagami Y (2016). Sarcopenia as a novel preoperative prognostic predictor for survival in patients with bladder cancer undergoing radical cystectomy. Ann Surg Oncol.

[25] Ohtaka A, Aoki H, Nagata M, Kanayama M, Shimizu F, Ide H (2019). Sarcopenia is a poor prognostic factor of castration-resistant prostate cancer treated with docetaxel therapy. Prostate Int.

